# PTBP3 promotes migration of non-small cell lung cancer through regulating E-cadherin in EMT signaling pathway

**DOI:** 10.1186/s12935-020-01240-9

**Published:** 2020-05-18

**Authors:** Qiong Wu, Bo Zhang, Ben Li, Xiang Cao, Xinming Chen, Qun Xue

**Affiliations:** 1 Department of Cardiothoracic Surgery, Affiliated Hospital of Nantong University, Nantong, China; 2 Medical School of Nantong University, Nantong, China

**Keywords:** PTBP3, NSCLC, EMT, Proliferation, Migration

## Abstract

**Background:**

Human polypyrimidine tract binding protein 3 (PTBP3), which belongs to the PTB family, demonstrate a significant tumorigenic capability in a variety of malignancies. However, the correlation between PTBP3 expression and pathogenesis of non-small cell lung cancer (NSCLC) remains little known. The design of the study attempts to examine the role of PTBP3 in the pathogenesis and prognosis of NSCLC.

**Methods:**

Our study conducted an investigation on the PTBP3 expression in human NSCLC tissues and a comprehensive analysis of the associations between three factors, involving the PTBP3 expression, clinicopathological features, and patient’s survival. Additionally, we also explored the role of PTBP3 expression in the proliferation and invasion of cancer cells.

**Results:**

The mining of The Cancer Genome Atlas (TCGA) database, western blotting and immunohistochemistry analyses showed significantly up-regulation of PTBP3 in NSCLC tissues than in normal tissues. Although overexpress or knockdown PTBP3 expression had no significant effect on proliferation of selected cell line, it could promotes migration of NSCLC cells via regulating E-cadherin in epithelial–mesenchymal transition (EMT) signaling pathway. Moreover, in the univariate analysis, the PTBP3-high is markedly related to poor overall survival results where hazard ratio (HR): 1.55; 95% confidence interval (95% CI): 1.87–2.01; p = 0.0001. Also, according to the multivariate analysis, an independent prognostic factor among NSCLC patients is the PTBP3 with an HR of 1.42 (CI: 1.09–1.9; p = 0.011). To explore potential signaling pathways, we used the TCGA dataset and performed Gene Set Enrichment Analysis (GSEA). Moreover, its expression in NSCLC was related to Tumor differentiation, lymph node metastasis, distant metastasis status and poor prognosis. Beside, by changing the expression of PTBP3 in selected cell lines, we found that overexpress or knockdown PTBP3 expression had no significant effect on proliferation, however it regulated migration possibly by EMT signaling.

**Conclusions:**

Collectively, our findings suggested that PTBP3 contributed to the progression of NSCLC and might serve as a potential target for anti-cancer therapy.

## Background

The most frequent cause of cancer-related deaths worldwide is lung cancer [1]. Generally, lung cancer is divided into approximately 85% non-small cell lung cancer (NSCLC) and the rest is small cell [2]. In spite of the rapid development of the treatments, NSCLC prognosis remains poor after surgical resection. The variation of the 5-year survival in lung cancer patients is dependent on the differences in stage and region. More specifically, it ranges from 14.7 to 21.4% [3]. Distant metastasis and long-term recurrence hinder the improvement of survival rate. Thus, identifying the molecular mechanisms underlying progression of NSCLC may help develop potential biomarkers and novel therapeutic targets for the malignancy.

EMT is considered to be activated in cancer cells, related to their isolation from primary tumors and their intravascular infiltration [4]. Loss of E-cadherin expression in EMT is considered to be extremely important, SNAI1 (also known as SNAIL), SNAI2 (SLUG) and SNAI3 (SMUC) are considered to be direct inhibitors of E-cadherin [5]. In the nascent fly mesoderm, snail promotes an E-cadherin to N-cadherin switch and initiates EMT [6]. Recent studies have shown that, snail is a potent trigger for EMT to allow NSCLC to invade and migrate [7, 8], c-Fos promotes tumor cell migration through EMT in head and neck squamous cell carcinoma [9].

Recent studies have shown that diverse RNA binding proteins (RBPs) are closely related to cancer process. Originally, PTB was recognized as an RNA-binding protein [10]. There are three members in the PTB family, namely the PTBP1 (PTB), PTBP2, and PTBP3. The PTBP2 is also known as nPTB or brPTB while the PTBP3 is also known as ROD1. Previous study showed PTBP3 protein level was increased in lung squamous cell carcinomas [11], breast cancer [12], and gastric carcinoma [13]. However, its expression and function in NSCLC remains unknown. Thus, the evaluation of PTBP3’s role in NSCLC continues to be vital. Our study conducted an investigation on the PTBP3 expression in human NSCLC tissues and a comprehensive analysis of the associations between three factors, involving the PTBP3 expression, clinicopathological features, and patient’s survival. Additionally, we also explored the role of PTBP3 expression in the proliferation and invasion of cancer cells. Our results showed that overexpress or knockdown PTBP3 expression had no significant effect on proliferation, however it regulated migration possibly by EMT signaling.

## Materials and methods

### RNA-sequencing patient data and bioinformatics analysis

We downloaded Level 3 RNA sequence data of NSCLC and corresponding clinical information from TCGA database. This included 108 normal samples and 1037 cancer samples.

### Tissue samples of patients

After surgical removal and maintenance at − 80 **°**C, we immediately froze the fresh samples of the tumorous and adjacent non-tumorous NSCLC for protein analysis. We obtained the NSCLC specimens from 147 patients for immunohistochemical analysis. Between 2013 and 2018, these patients did not undergo any radiotherapy or chemotherapy before having their surgical resection procedure done at the Affiliated Hospital of Nantong University. We obtained the signed informed consent from all 147 patients. The Ethics Committee of the Affiliated Hospital of Nantong University approved the use of these specimens and data for our research.

### Gene set enrichment analysis

Based on the TCGA dataset, we executed the enrichment analysis of the gene set to further explore the biological functions of PTBP3. Initially, these patients were classified into high- and low-PTBP3 groups. For each analysis, we repeatedly completed the gene set permutations for 1000 times. The phenotype label that we used was the expression level of PTBP3. To screen the significant signaling, we set the *P* value limit at < 0.05.

### Western blot analysis

Using the Radio Immunoprecipitation Assay (RIPA) buffer (high) (R0010; Solarbio Life Sciences, Beijing, China), we extracted the total tissue protein according to the guidelines set by the manufacturer. Then, we adopted a Bio-Rad protein assay (Bio-Rad, Hercules, CA) to calculate protein concentrations. We worked with the SDS-PAGE to separate the protein lysates for all samples before moving them to polyvinylidene fluoride (PVDF) microporous membrane (Immobilon, Millipore). Furthermore, in Tris-Buffered Saline-Tween (TBST), we blocked the membranes for 2 h with 5% skim milk (Biosharp, China) at room temperature. Afterward, using anti-PTBP3 (1:1000 dilution; Proteintech), anti-GAPDH (1:10,000 dilution; Proteintech), we incubated the membranes overnight at 4 **°**C. With Goat anti-Mouse/Rabbit (1:10,000 dilution; Li-cor), we washed the membranes three times and proceeded with their incubation at room temperature for 2 h. Finally, the ECL detection systems (Pierce, Rockford, IL, USA) identified the protein bands while the Image J software (National Institutes of Health, Bethesda, MD, USA) analyzed the intensity of the bands.

### Immunohistochemistry

The sample was fixed using 10% formalin before embedding. The embedded paraffin was then prepared into four-micrometer-thick tissue sections. After the tissue sections were baked in a 60 °C incubator for 6 h, the sections were dewaxed in xylene, hydrated and subjected to antigen heat repair by high pressure, followed by blocking of endogenous peroxidase activity in the tissue with 3% hydrogen peroxide and then incubated with a polyclonal antibody against PTBP3 (1:100 dilution; Proteintech) for 2 h at room temperature. Washing three times with PBS for 5 min, use the corresponding secondary antibody to incubate the tissue sections at room temperature for 1 h. We visualized the tissue sections through the DAB. Then, we applied hematoxylin to counterstain the tissue section before dehydrating and sealing it with neutral resin.

### Immunohistochemical evaluation

Two investigators evaluated PTBP3 immunostaining results in all sections. The intensity of staining was scored as follows: 0 (no staining), 1 (light yellow), 2 (deep yellow),and 3 (brown particles). The percentages of stained cells were scored as follows: 1 (1–30%), 2 (31–50%), 3 (51–70%), or 4 (71–100%). Then, the two scores are multiplied before being grouped into two PTBP3 expressions, known as the low-level with a score of ≤ 4 and high-level with a score of > 4.

### Cell culture and transfection

The NSCLC cell lines Shanghai Cell Bank (Shanghai, China) we purchased include the A549, H1299, and SPCA-1. Then, using RPMI 1640 medium (Gibco, USA) at 37 **°**C with 5% CO_2_, we cultured these cell lines that contain 10% qualified fetal bovine serum (FBS). The cells used in the experiments were in the third to sixth generation. LV-PTBP3 and LV-PTBP3-RNAi targeted at PTBP3 were designed and synthesized by Genechem (Shanghai, China). Transfections were performed to decrease PTBP3 expression using shRNA (targeting sequence:CCGGCAGAGACTTCACTCGCTTAGACTCGAGTCTAAGCGAGTGAAGTCTCTGTTTTTG). CON238 and CON313 were used as control. We abided by the manufacturer’s instructions to perform the transfection.

### Cell Counting Kit 8 Assays

To evaluate cell proliferation, we utilized the cell counting kit-8 (CCK8) assays. At a density of 1 × 10^3^ cells/well, we seeded the cells into the 96-well cell culture cluster plates. Then, in each well, we added the CCK-8 reagents before incubating the wells at 2 h at 37 **°**C in the dark. Using a microplate reader (Bio-Rad, USA), we measured the absorbance value at 450 nm. We conducted repetitions of the experiments for at least three times.

### Cell cycle analysis

Through centrifugation, we collected the cells from 6-well plates before washing them in PBS. Then, at − 20 **°**C, we fixed the cells with 70% ethanol overnight. Then, the cells were incubated with 500 uL PI/RNAse solution at 37 °C for 30 min. Acquired and analyzed datas using Attune NxT flow cytometer.

### Invasion and migration assays

We put the 8 μm pore size Transwell filter chamber inserts (Corning, NY, USA) to work by examining cell migration and invasion assay. After transfected with the indicated virus, 2 × 10^4^ cells were respectively seeded into transwell chambers. To fuel the migration or invasion, we added the RPMI-1640 containing 10% FBS to the lower chamber. For 24 h at 37 **°**C and 5% CO_2_, we incubated the migrated cells. Then, we fixed the migrated cells in 4% paraformaldehyde and stained them with 0.1% crystal violet and imaged before taking an image.

### Assay with a wound-healing property

We performed cell cultivation in 6-well culture plates. Then, we grew them to 80% confluence. Using a 200-µL pipette tip, we scratched the confluent cells before proceeding with the PBS buffer to wash and rid them of cell debris. The selected wound closure area was observed and photographed at different times.

### Statistical Analysis

R software was utilized to perform bioinformatics analysis based on TCGA data. We performed our statistical analyses through the SPSS 24.0 software. Then, we evaluated the associations between the PTBP3 expression and clinicopathological features through the Chi square (χ^2^) test. We used the logistic regression model to confirm the correlation between PTBP3 expression and lymph node metastasis. We set our statistical significance for differences with P-values of < 0.05.

## Results

### PTBP3 was overexpressed in NSCLC tissues

**Fig. 1:**
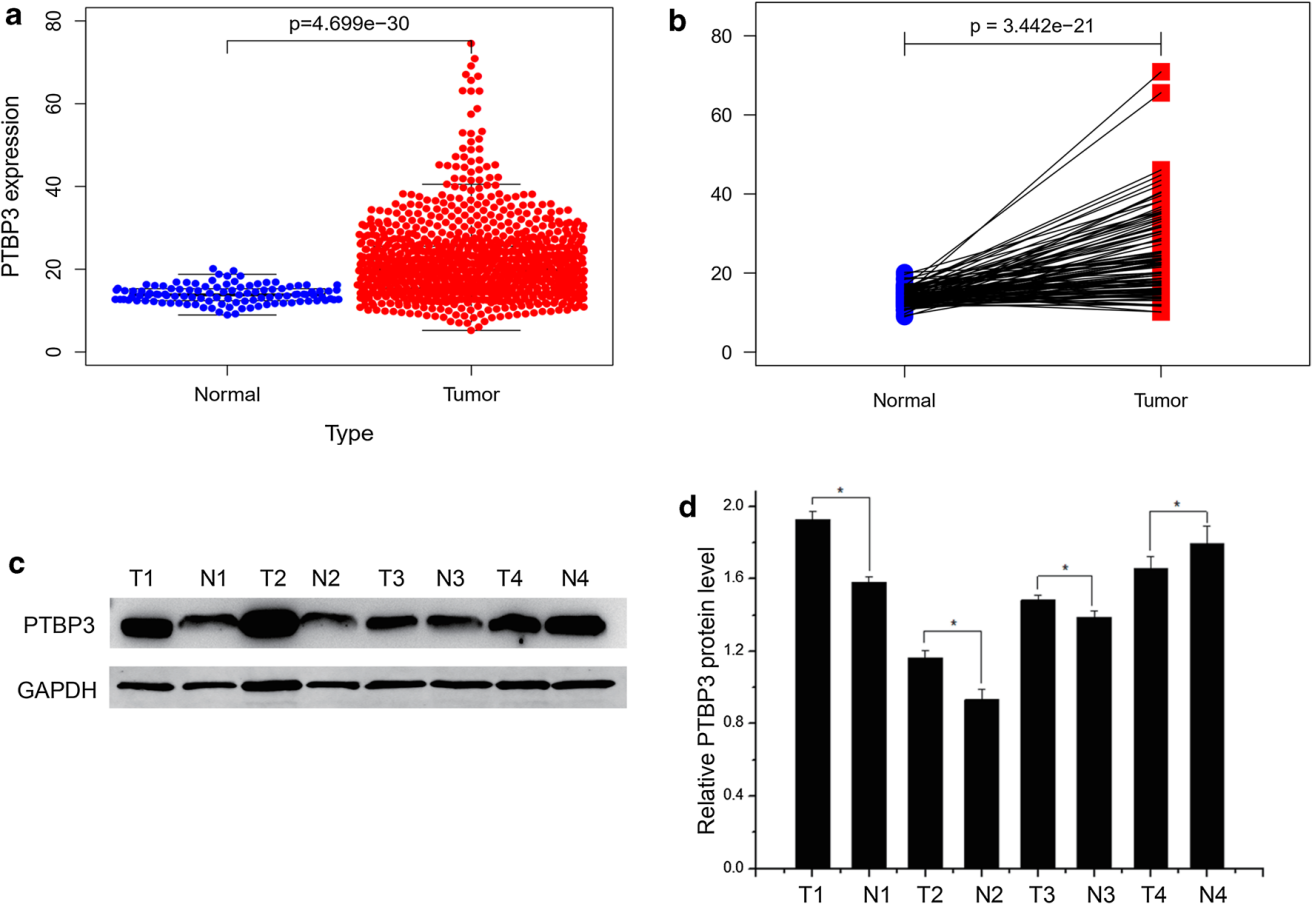
PTBP3 expression in NSCLC tissues. **a** PTBP3 expression between NSCLC tissues and normal tissues. **b** PTBP3 expression between NSCLC tissues and their adjacent nontumorous lung tissues. **c**, **d** Using the Western blot analysis, we obtained results that highlighted PTBP3 expression levels in 147 paired fresh lung cancerous tissues (T) and adjacent non-tumorous lung tissues (N). Then, we based our analysis of the relative quantification on grayscale values

In contrast with the normal tissues, the significant upregulation of PTBP3 in NSCLC tissues originated from the mining of the TCGA database (Fig. 1a, b). We also perform western blotting based on obtained 147 pairs of NSCLC samples, and reconfirmed the similar expression tendency of PTBP3 between cancer and normal tissues (Fig. 1c, d).

### Relationship between PTBP3 expression and clinicopathological parameters in NSCLC

**Fig. 2:**
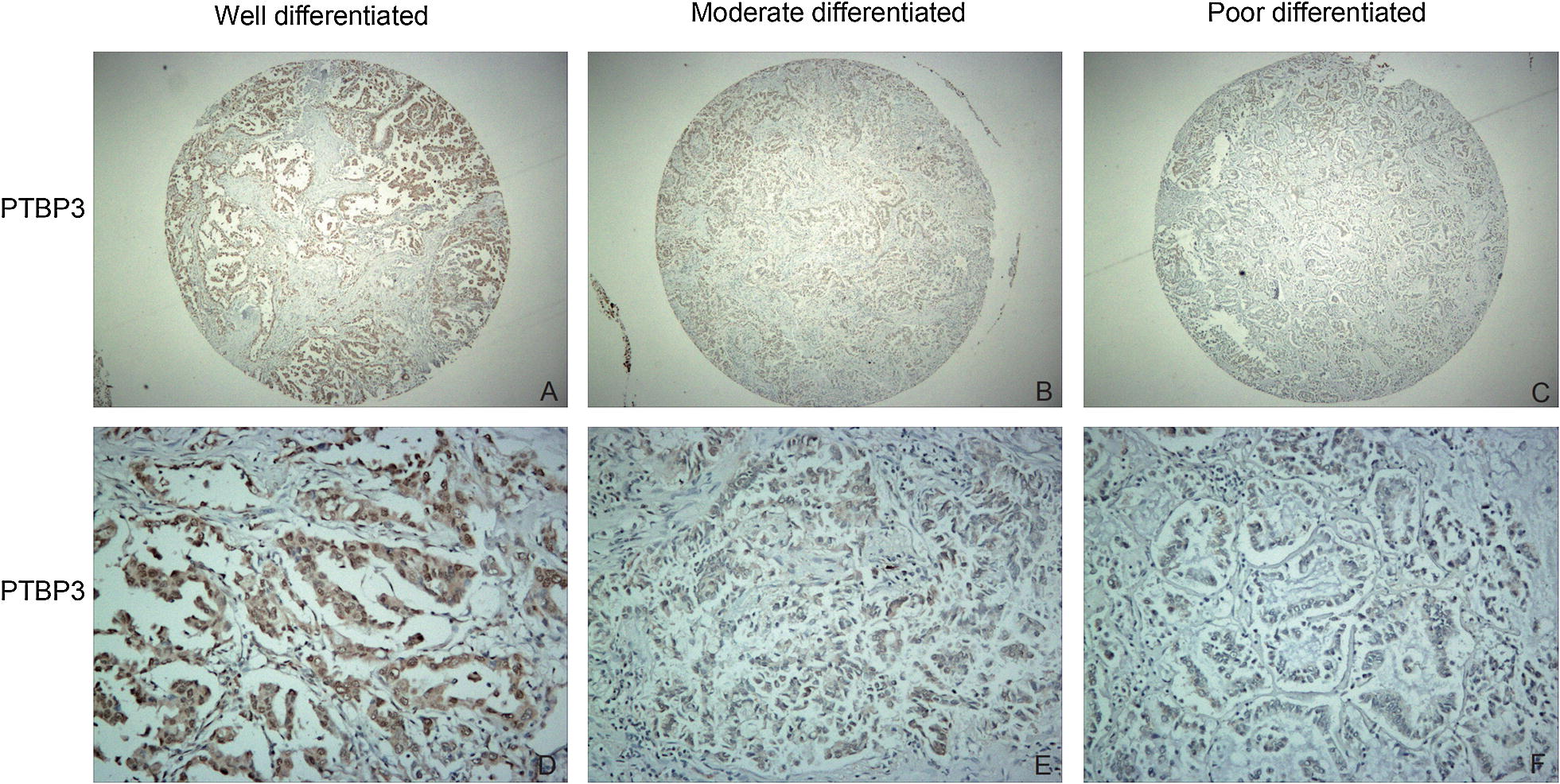
Immunohistochemical staining for PTBP3 expression in NSCLC. We used a PTBP3 antibody to stain tissue sections and hematoxylin to counterstain them. In NSCLC, the high expression level of the PTBP3 was evident. The samples were divided into well-differentiated (**a**, **d**), moderately differentiated (**b**, **e**), and poorly differentiated (**c**, **f**) (A, B, C ×40 magnification; **d**–**f** ×200 magnification)

A total of 147 NSCLC patients were analyzed by immunohistochemistry to study the expression of PTBP3. As is shown in Table 1, 75 sections were identified as low-level PTBP3 (51.02%) expression yet 72 samples revealed high-level PTBP3 (48.98%) expression. The positive correlation of the PTBP3 expression is present with three factors: First is with tumor differentiation (P = 0.003), second with lymph node metastasis (P = 0.004), and third with distant metastasis status (P = 0.002). However, the correlations between PTBP3 expression and gender (P = 0.671), age (P = 0.884), smoking status (P = 0.217), tumor size (P = 0.292) and tumor classification (P = 0.082) were not statistically significant (Table 1). The expression of PTBP3 was up-regulated in poorly differentiated specimens compared to highly differentiated and moderately differentiated specimens (Fig. 2).

**Table 1:** Correlationship between PTBP3 expresstion and NSCLC patient clinicopathological parameters

Clinicopathological parameters	Total	PTBP3		*P*-value
		Low (n = 75)	High (n = 72)	
Gender				
Male	70	37	33	0.671
Female	77	38	39	
Age (years)				
< 60	56	29	27	0.884
≥ 60	91	46	45	
Smoking status				
Smoker	46	20	26	0.217
Nonsmoker	101	55	46	
Tumor size (cm)				
≤ 3	80	44	36	0.292
> 3	67	31	36	
Tumor classification				
T1 + T2	96	54	42	0.082
T3 + T4	51	21	30	
Tumor differentiation				
Poor-Moderate	97	41	56	0.003*
Well	50	34	16	
Lymph node metastasis				
No	89	54	35	0.004*
Yes	58	21	37	
Distant metastasis status				
M0	124	70	54	0.002*
M1	23	5	18	

* P < 0.05 was considered significant

### Expression of PTBP3 in the prognosis of lymph node metastasis

Studies have shown that lymph node metastasis will further aggravate the severity of lung cancer patients and is closely related to the survival rate of patients [14]. In our study, we found that PTBP3 overexpression in NSCLC tissues were correlated with tumor differentiation, lymph node metastasis and distant metastasis status. So we further analyzed whether PTBP3 can be used as a risk factor for lymph node metastasis. The univariate analysis showed that tumor size and PTBP3 expression were associated with lymph node metastasis (Table 2).

**Table 2:** Risk factors for lymph node metastasis in patients with NSCLC

Characteristics	Univariate analysis			
	B	SE	95% CI	P-value
Age	− 0.573	0.520	0.203–1.564	0.271
Gender	1.211	0.661	0.919–12.272	0.067
Smoking status	− 0.395	0.808	0.138–3.279	0.625
Tumor size (cm)	1.362	0.549	1.332–11.455	0.013*
Tumor differentiation	0.061	0.162	0.773–1.461	0.709
PTBP3 expression	1.047	0.477	1.118–7.267	0.028*

* Statistical analyses were performed by the Logistic regression analysis, and P < 0.05 was considered significant

### Poor prognosis of NSCLC patients correlated with high expression of PTBP3

**Fig. 3:**
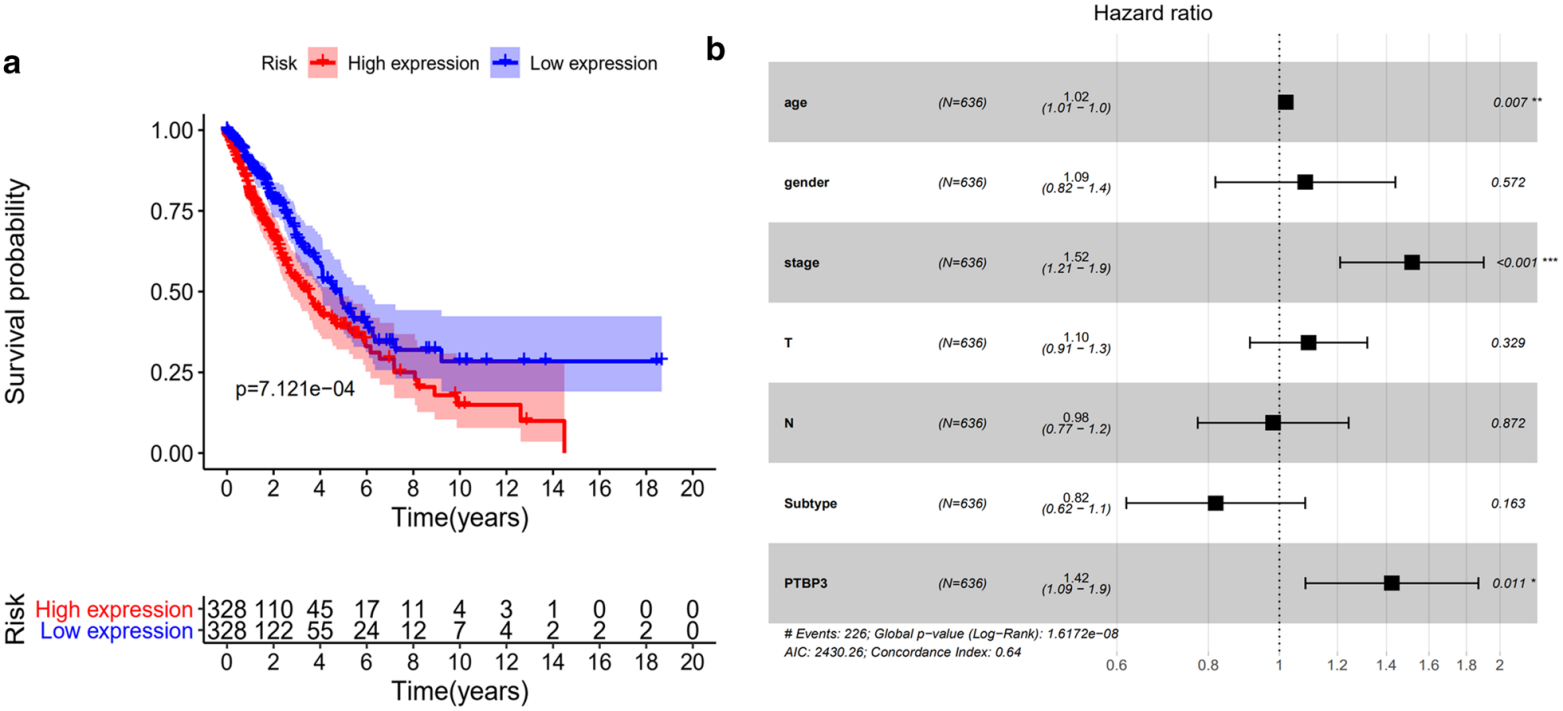
The expression levels of PTBP3 have a correlation with the overall survival in patients with NSCLC. **a** Utilized TCGA data to conduct survival analysis. **b** An independent prognostic factor for NSCLC patients is PTBP3, which is determined through the multivariate analysis

Additionally, the results from our study suggest the evidential association between PTBP3 expression and survival of patients with NSCLC. The survival curves constructed based on TCGA database revealed that higher PTBP3 expression displayed shorter Overall survival (OS) compared with lower PTBP3 expression in NSCLC patients (Fig. 3a). The univariate analysis revealed that PTBP3-high markedly related to a poor overall survival (HR: 1.55 95% CI: 1.87–2.01; p = 0.001). Using a multivariate analysis, we found that an independent prognostic factor for NSCLC patients is the PTBP3. In this case, PTBP3 presents values with an HR of 1.42 (95% CI: 1.09–1.9; P = 0.011; Fig. 3b).

### GSEA identifies PTBP3-related signaling pathways

**Fig. 4:**
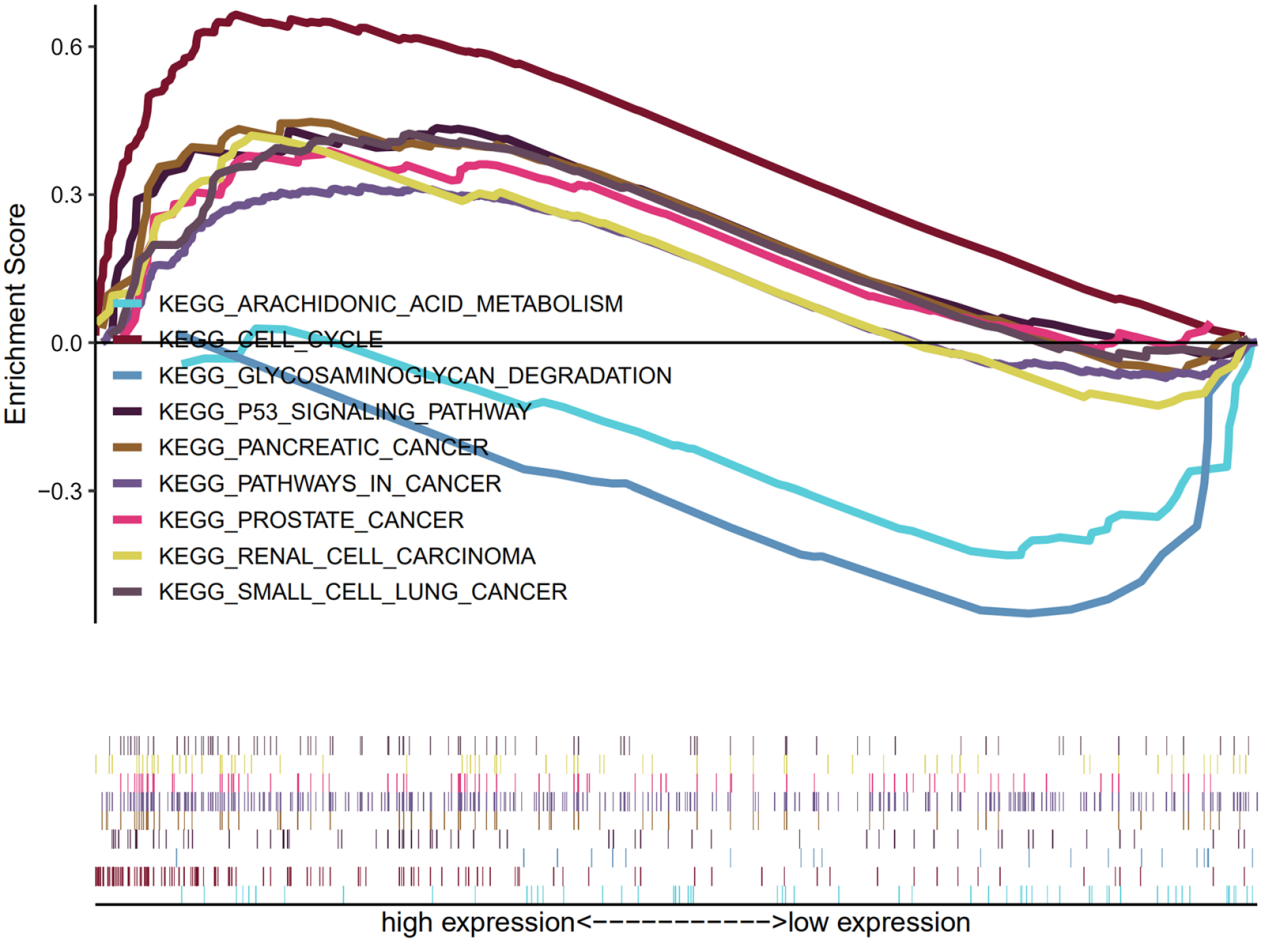
GSEA identifies PTBP3-related signaling pathways

To conduct the GSEA analysis, we adopted the expression data sets between low and high PTBP3, which revealed results that identified signaling pathways that are differentially activated in NSCLC. Then, we based our identification of the most significantly enriched signaling pathways on their normalized enrichment score (NES) (Fig. 4). Our results confirmed the differential enrichment of the pathways, namely P53 signal, cancer, cell cycle, and pancreatic cancer, in PTBP3 high expression phenotype.

### NSCLC cell lines selection and transfection efficiency

**Fig. 5:**
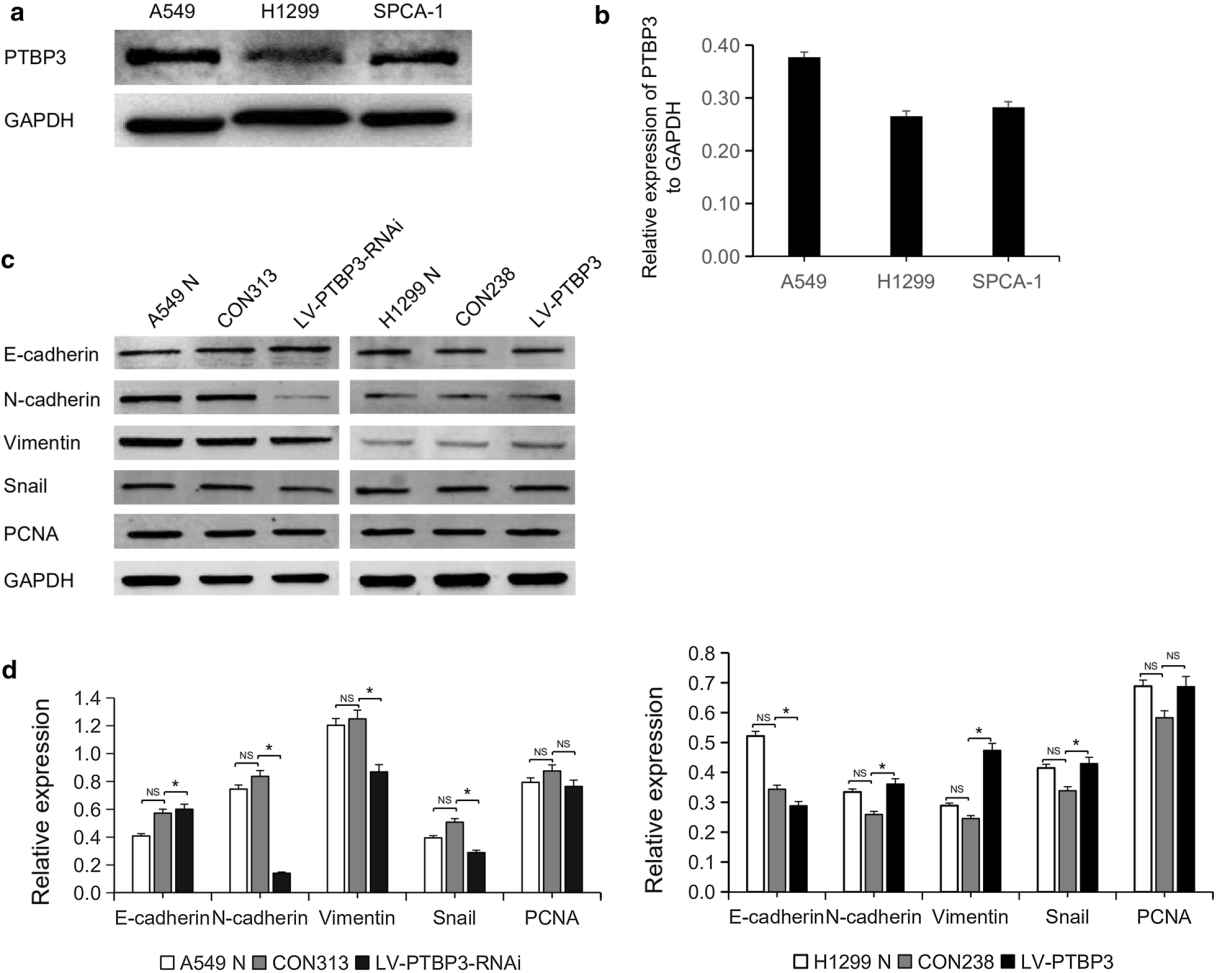
PTBP3 expressions and markers that are EMT-related in NSCLC cell lines. **a**, **b** The Western blotting analysis results illustrated the PTBP3 expression in three NSCLC cell lines. **c**, **d** The Western blotting analysis results identified the expressions of the markers associated with EMT in the A549 N (untreated group), such as the E-cadherin, N-cadherin, vimentin, SNAIL, and PCNA. CON313 (negative control group), LV-PTBP3-RNAi (interference group), H1299 N (untreated group), CON238 (negative control group), LV-PTBP3 (overexpression group) (*NS* non-significant, *P < 0.05)

**Fig. 6:**
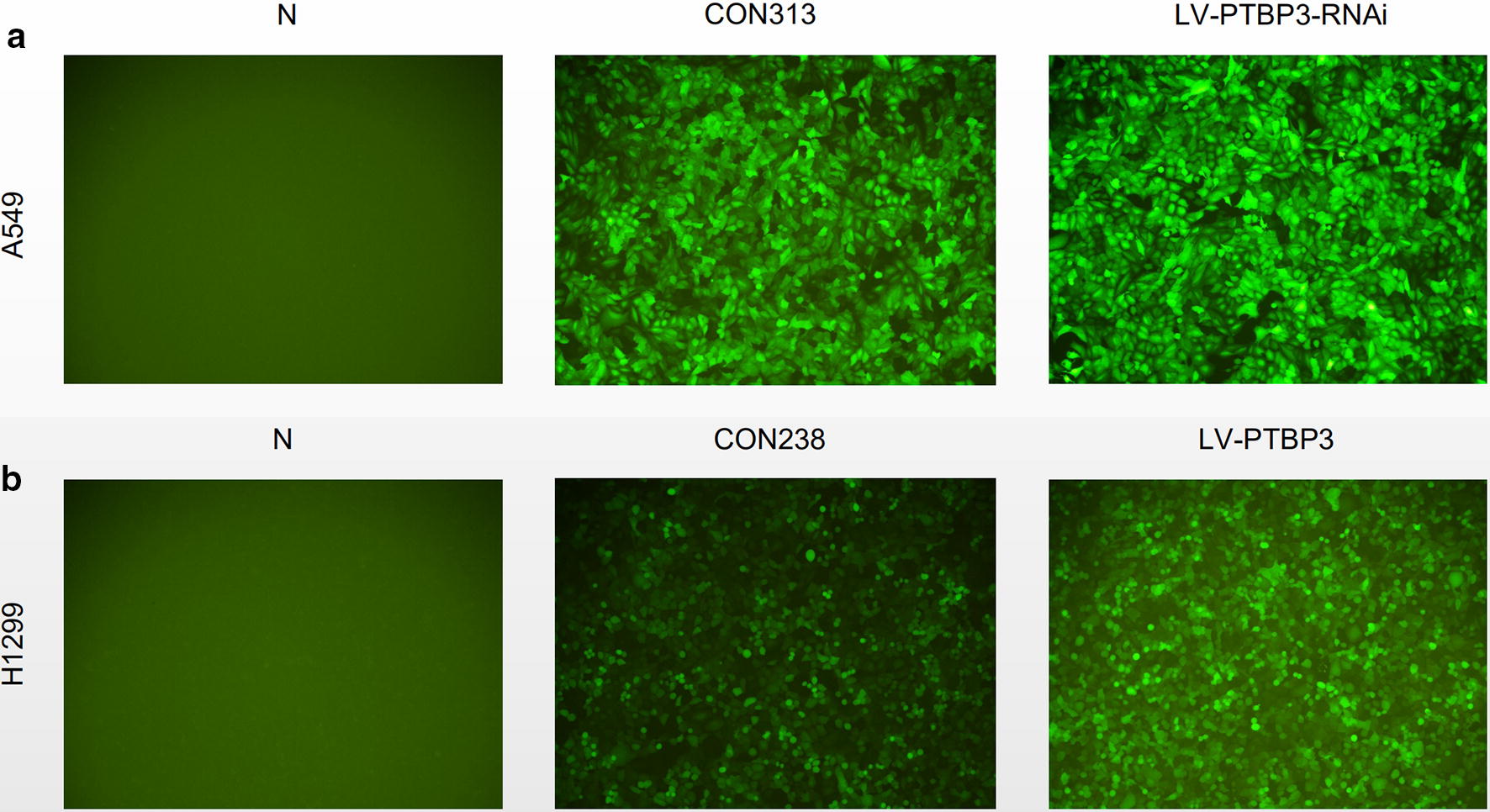
The efficiency of lentivirus infection in NSCLC cells. **a** A549, **b** H1299 Observed infection efficiency after 72 h of corresponding lentivirus infection

The Western blotting results illustrated the PTBP3 expression in NSCLC cell lines. Additionally, the results confirmed that in A549 cells, PTBP3 had higher expression and a lower expression in H1299 cells (Fig. 5a, b). Therefore, we decided that A549 cells were transfected by LV-PTBP3-RNAi and LV-PTBP3 was used for H1299 cells transfection. Transfection efficiency was shown in Fig. 6.

### Effect of PTBP3 on proliferation of NSCLC cells

**Fig. 7:**
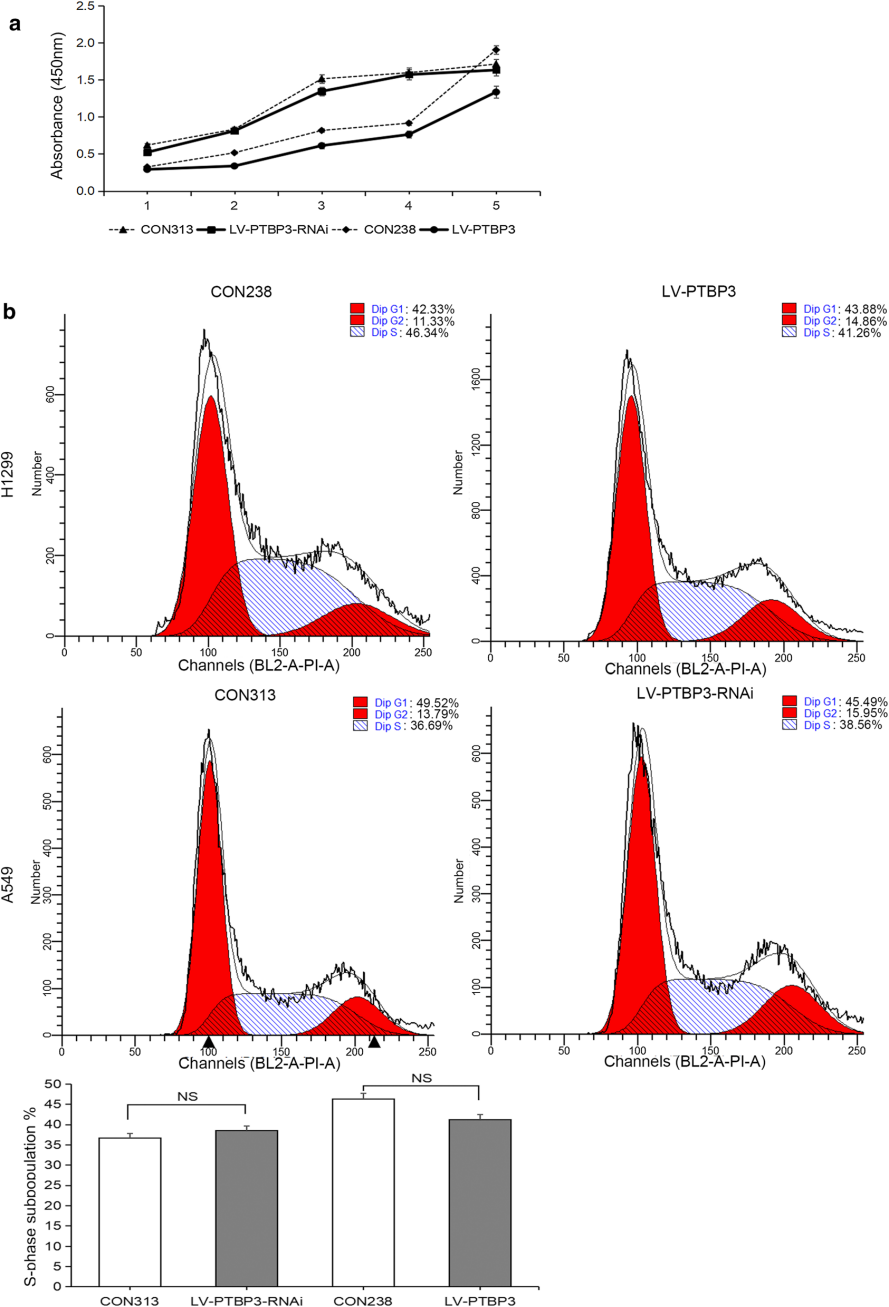
Effect of PTBP3 on proliferation of NSCLC cells. **a** CCK-8 assays were performed to analysis NSCLC cells proliferation. **b** Flow cytometry was performed to evaluate the effects of PTBP3 expression on the G1/S phase transition

We put the CCK-8 assays and cell cycle analysis into action to investigate the role of PTBP3 on proliferation of NSCLC cells. The results confirmed no statistical significance between interfered or overexpressed PTBP3 cells and their corresponding control cells (Fig. 7), indicating that PTBP3 had no significant effect on proliferation in vitro.

### Effect of PTBP3 on migration and invasion of NSCLC cells

**Fig. 8:**
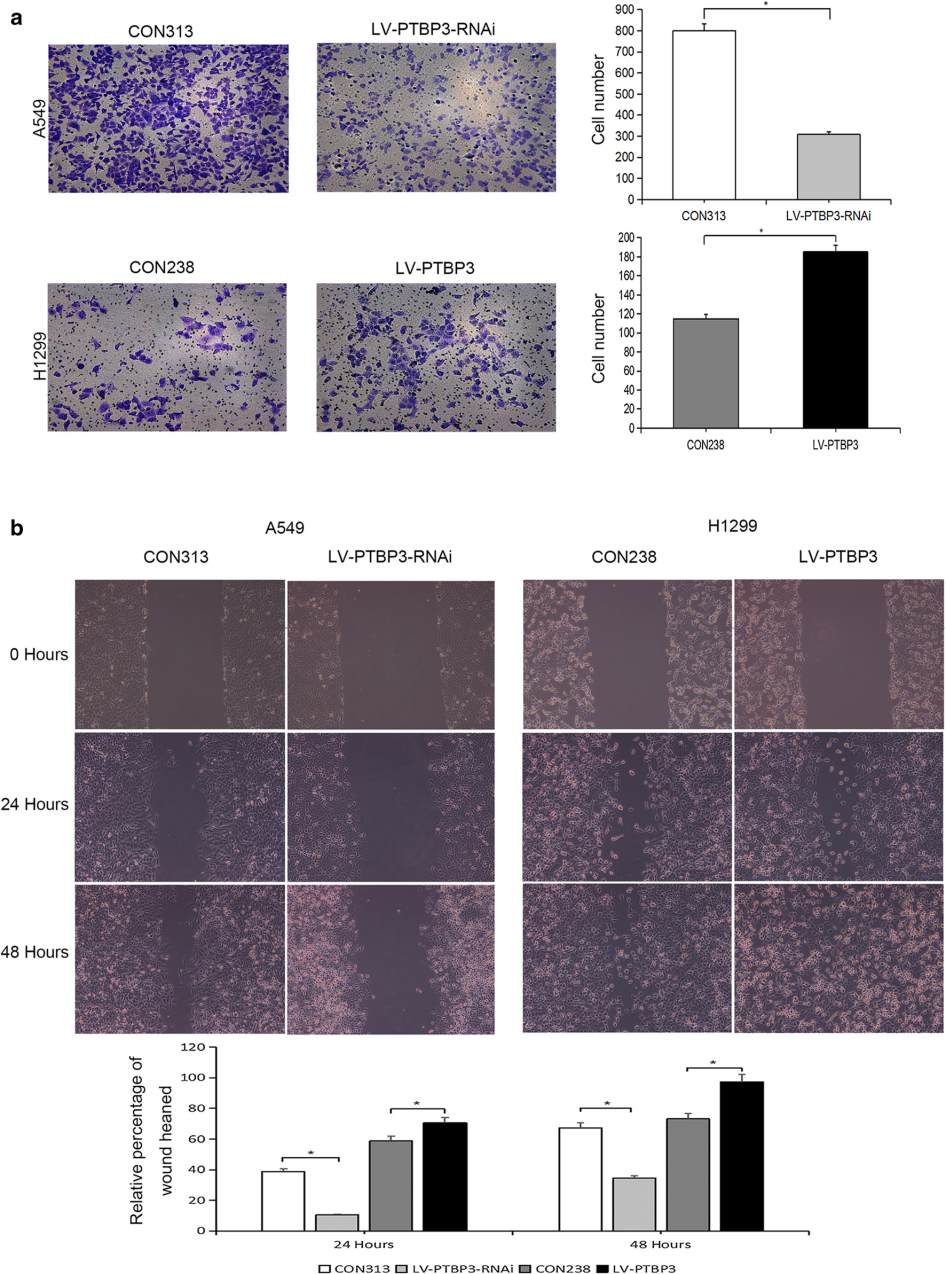
Effect of PTBP3 on migration and invasion of NSCLC cells. **a** Transwell migration and invasion and **b** wound-healing assays were conducted after infected with the lentivirus to evaluate the effects of PTBP3 on migration and invasion of NSCLC cells

**Fig. 9:**
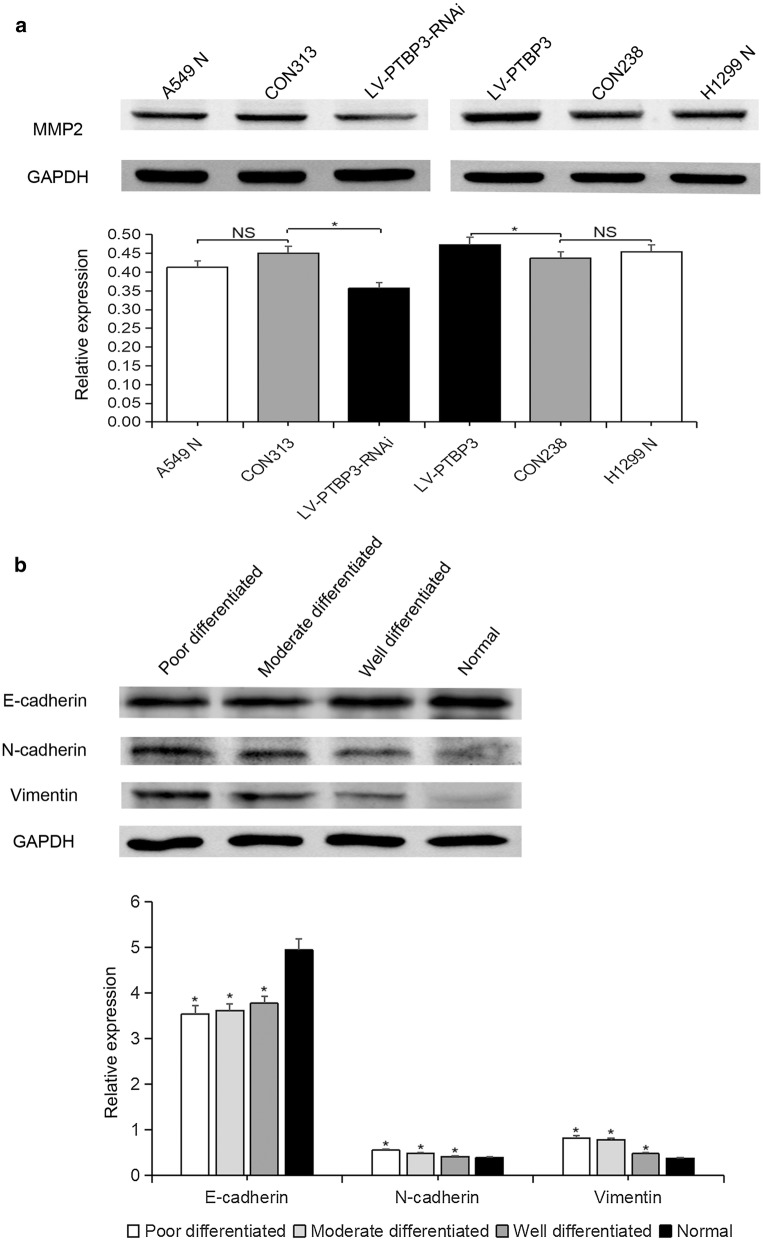
The effect of changes in PTBP3 expression on MMP2 and the association between EMT-related molecular markers and tumor differentiation. **a** The Western blotting analysis results illustrated the MMP2 expression in the A549 N (untreated group), CON313 (negative control group), LV-PTBP3-RNAi (interference group), H1299 N (untreated group), CON238 (negative control group), LV-PTBP3 (overexpression group). **b** The Western blotting analysis results identified the expressions of the markers associated with EMT in tumor tissue with different degrees of differentiation and normal lung tissue, such as the E-cadherin, N-cadherin and vimentin. (*NS* non-significant, *P < 0.05)

We proceeded with our investigative approach on the impact of the PTBP3 expression on the NSCLC cells in terms of invasion and migration. Then, to examine the capabilities of the cell to migrate and invade, we applied the Transwell and wound-healing assays. The results of the experiment showed that in H1299 cells, overexpression of PTBP3 was significantly increased in invasiveness compared with the control group. Accordingly, in A549 cells, the invasiveness was significantly reduced as compared with the control group after interfering with PTBP3 expression (Fig. 8). When we overexpressed or inhibited PTBP3 in H1299 or A549 cells, this either enhanced or inhibited the ability of the cells to migrate and invade. Similarly, we examined changes in MMP2 expression after interference or overexpression of PTBP3 by WB, and the results confirmed our previous conclusions (Fig. 9a).

### PTBP3 promotes NSCLC metastasis via EMT pathway

One of our research goals is to conduct an analysis of the epithelial–mesenchymal transition (EMT)-related proteins and their expressions, such as after the overexpression or interference with PTBP3 in NSCLC cells. WB results showed that after interference with PTBP3 expression, E-cadherin was up-regulated, while N-cadherin, vimentin, and snail decreased. Similarly, after overexpression of PTBP3, E-cadherin expression decreased, while N-cadherin, vimentin, and snail expression were up-regulated (Fig. 5c, d). Therefore, we consider that PTBP3 initiates the EMT pathway and promotes the invasion and migration of NSCLC by down-regulating the expression of E-cadherin. In spite of the PTBP3 expression’s interference or overexpression, the expression of the proliferating marker PCNA had no significant difference. This also validates the results of previous CCK8 and cell cycle experiments. There was no significant difference in expression between the untreated group (A549N, H1299N) and the negative control group (CON313, CON238), indicating that the lentiviral vector had no significant effect on NSCLC cells (Fig. 5c, d). In addition, we used WB to analyze the relationship between tumor differentiation and EMT-related molecular markers (Fig. 9b). Results showed that E-cadherin increased with the increase of tumor differentiation, while N-cadherin and vimentin decreased. This verifies our previous conclusions.

## Discussion

Although various molecule mechanisms were investigated in NSCLC, improvement in prognosis of this disease was not effective enough [16]. So, Researching potential target for anti-cancer therapy to ameliorate the prognosis of NSCLC is urgently needed. PTBP3 has been identified as a regulatory protein involved in cell differentiation since its discovery [17].

Recent literature acknowledges the close relationship of PTBP3 with the progression of different malignant tumors. Previous study showed PTBP3 protein level was increased in lung squamous cell carcinomas [11], breast cancer [12], and gastric carcinoma [13]. Interestingly, however, PTBP3 expressed in glioblastoma multiforme less than normal brain tissue [15]. Our study affirmed the overexpression of PTBP3 in patients with NSCLC. We considered that the abnormal expression of PTBP3 in the T4N4 group was caused by individual patient differences. Clearly, PTBP3 plays a potential role in the occurrence and development of NSCLC.

Recently, it has been reported that PTBP3 regulates the expression of the transcription factor ZEB1 by stabilizing the 3’UTR of ZEB1, preventing its degradation. Consequently, this stimulates the migration and invasion of the breast cancer cells, as well as the mesenchymal transition of the epithelial cell. In breast cancer patients, lymph node metastasis, histological grade, TNM staging, and poor prognosis are all associated with high levels of PTBP3 expression [18]. MiR-210 promotes proliferation and inhibits apoptosis of glioblastoma multiforme cells by decreasing the expression of PTBP3 [15]. However, in gastric cancer, PTBP3 promotes metastasis of gastric cancer by regulating CAV1 [13] and reduces the sensitivity of gastric cancer cells to 5-Fu by the HDAC6/Akt/TYMS pathway [19].

Our study confirms the positive correlation of the overexpression of PTBP3 in NSCLC tissues with a variety of clinicopathological parameters. These parameters include the differentiation of tumor, metastasis of lymph node, and the status of distant metastasis. We verified the association of the tumor size and PTBP3 with the metastasis of the lymph node through the univariate analysis. Equally important is that the upregulation of the PTBP3 expression is linked to poor prognosis of NSCLC patients. In light of the ability of epithelial-derived malignant cells to migrate and invade, a relevant and vital biological process is called the EMT. The main features of EMT are the reduction of cell adhesion molecules (such as E-cadherin), and the upregulation of N-cadherin and Vimentin [20]. The epithelial phenotypes of the epithelial cells, such as cell polarity and connection to the basement membrane, are lost through the EMT. Additionally, these cells achieve migration and invasion, which are higher interstitial phenotypes [21]. Overexpression of PTBP3 promotes breast cancer cell proliferation and epithelial mesenchymal transition (EMT) [18]. Whether PTBP3 promotes NSCLC metastasis by activating EMT signaling pathway remains unknown. Our results suggest that PTBP3 may initiate EMT signaling via down-regulation of E-cadherin expression and serve as an oncogene in the development of NSCLC. Beside, detection of CCK-8, cell cycle assays and molecular markers related to cell proliferation showed that PTBP3 had no significant effect on proliferation of NSCLC cells. Interestingly, PTBP3 promotes tumor migration and invasion by mediating CAV1 in gastric cancer, but overexpression/knockdown of PTBP3 expression also has no effect on proliferation [13].

## Conclusions

In conclusion, elucidating the molecular mechanism of EMT process in NSCLC, and exploring the diagnostic methods and treatment methods based on EMT molecules are the key issues of research. The molecular mechanism of PTBP3 in human malignancies is still unclear. The way PTBP3 plays a role in human malignancies appears to be tissue specific. Our results suggest that PTBP3 may be an oncogene that promotes NSCLC metastasis.

## Data Availability

Data sharing not applicable to this article as no data-sets were generated or analysed during the current study.

## Abbreviations

PTBP3: Human polypyrimidine tract binding protein 3
NSCLC: Non-small cell lung cancer
EMT: Epithelial–mesenchymal transition
TCGA: The Cancer Genome Atlas
95% CI: 95% confidence interval
HR: Hazard ratio
GSEA: Gene Set Enrichment Analysis
RBPs: RNA binding proteins
RIPA: Radio Immunoprecipitation Assay
PVDF: Polyvinylidene fluoride
TBST: Tris-Buffered Saline-Tween
CCK8: Cell Count Kit 8
FBS: Fetal bovine serum
OS: Overall survival
NES: Normalized enrichment score
WB: Western blotting
